# Neonatal mortality in the case of Felege Hiwot referral hospital, Bahir Dar, Amhara Regional State, North West Ethiopia 2016: a one year retrospective chart review

**DOI:** 10.1186/s13052-018-0498-5

**Published:** 2018-05-21

**Authors:** Tilahun Tewabe, Yenatfanta Mehariw, Eyerusalem Negatie, Bertukan Yibeltal

**Affiliations:** College of Medicine and Health Sciences, Bahir Dar University, Bahir Dar, Ethiopia

**Keywords:** Neonatal mortality, Felege Hiwot referral hospital, Bahir Dar, Ethiopia

## Abstract

**Background:**

Ethiopia is among the countries with the highest neonatal mortality with the rate of 37 deaths per 1000 live births. In spite of many efforts by the government and other partners, non significant decline has been achieved over the last 15 years. Thus, identifying the prevalence and associated factors of neonatal mortality is very crucial for policy and program improvement. This study was designed to assess neonatal mortality rate in Felege Hiwot referral hospital, North West Ethiopia.

**Methods:**

A hospital based chart review was done in Felege Hiwot referral hospital based on patient charts from July 2015 to June 2016. The data were collected using structured checklists. The collected data was coded, filtered and entered in to Microsoft Excel 2007 and transferred to STATA version 12.0 for analysis. Binary logistic regression analysis was used to identify factors associated with neonatal mortality. A p - value of < 0.05 was considered as significant.

**Results:**

The prevalence of neonatal mortality in Felege Hiwot referral hospital was 13.29% (95% CI: 10.09–17.07). Early age of the newborn (< 7 days) [AOR = 0.39 (0.16–0.97)], gestational age at delivery [AOR = 2.14 (1.0–4.52)], late initiation of breastfeeding [AOR = 2.89 (0.99–8.38)], non exclusive breastfeeding [AOR = 6.77 (3.04–15.07)], inadequate ante natal visit [AOR = 5.02 (1.02–24.70)] were the determinant factors for neonatal death.

**Conclusions:**

This study revealed that neonatal mortality is still high in the study area. Early age of the newborn, late initiation of breastfeeding, exclusive breastfeeding and ante natal vist were the determinant factors for neonatal mortality in the study area. Therefore, giving attention for newborns who are small for age, timely initiation of breastfeeding, exclusive breastfeeding and increasing ante natal visit were recommended to reduce neonatal mortality.

## Background

Globally, 6.6 million kids died before celebrating their fifth birthday per year. About 5 million deaths occurred within the first 28 days of life. This showed nearly 44% of under five mortalities and 60% of infant mortalities are covered by neonatal deaths. Above 98% of neonatal mortalities occurred in developing countries. Sub Saharan Africa takes the highest rate of newborn death, these are regions having least improvement in decreasing neonatal death rates [1].

In Ethiopia there is a high prevalence neonatal mortality. The trends of neonatal mortality in the country has slight decrease over past 20 years. Which were 46 in 1991–1995, 42 in 1996–200, 39 in 2001–2005 and 37 in 2006–2011 per 1000 live births. In spite of this, around 63% of infant mortalities in the country happened within the first 28 days of newborns life [2].

Most cases of neonatal deaths i.e., 99% occurred in low and middle income countries. Around half of the cases occurred among home deliveries, making the global rate of neonatal mortality 30 per 1000 live births [3].

Reducing this huge number of neonatal death is a major challenge in Ethiopia since the targeted health interventions proposed to cover most fatal causes of neonate are usually vary from those required to tackle under five mortality [2].

Neonatal mortality rate has shown slow decline i.e. by 0.9% per year from 1995 to 2010. This high prevalence of early neonatal death comprises 74% of neonatal deaths [2, 4]. And a study done in Jimma showed that neonatal mortality rate was 35.5 per thousand live births [5] and 14.4% in Addis Ababa [6].

This number is higher than former countries with high prevalence of neonatal deaths such as India and Indonesia. This obviously showed the condition of neonatal deaths is still towering and non progressing telling targeted interventions with all partners at various levels [7, 8].

Therefore, the study was intended to assess neonatal mortality in Felege Hiwot Referral Hospital from July 2015 to July 2016.

## Methods

### Study design and setting

Institution based retrospective chart review was conducted in Felege Hiwot referral hospital based on patient charts from July 2015 to June 2016**.**

The study was conducted in Felege Hiwot Referral Hospital, Bahir Dar, North West Ethiopia. It is located 563 Kms far from Addis Ababa. Felege Hiwot referral hospital officially commenced its function in 1963 and currently it delivers health care services with medical, surgical, gynecological, orthopedic, intensive care units, paediatrics and ophthalmological wards with a total of 375 beds and 561 staffs. Annually, nearly 6300 neonates were seen with different health problems. The neonatal unit has 60 beds, 5 pediatricians and 20 nurses.

The sample size was calculated with single population proportion formula and by taking into consideration: prevalence (P) of neonatal mortality 43.8% [9], confidence level (CL) 95%, margin of error (d) 5, 10% non response rate and by using simple random sampling technique a total of 410 neonatal charts were selected for study.

### Measurement

Data collection was done by using checklists which were prepared by using similar studies done on related topics [4, 5, 9]. Which consists: socio demographic information, risk factors of neonatal deaths and health service utilization characteristics. Eight data collectors and two supervisors were participated in the data collection. Before the actual data collection started, training was given for data collectors and supervisors for 1 day about proper data collection and recording.

### Data analysis

The collected data from patient charts were coded, filtered and entered in to Microsoft excel 2007 and transferred to STATA Version 12.0 for analysis. To identify factors associated with neonatal mortality, first bivariate analysis was done to each independent variable with the dependent variable. Those variables which were associated with neonatal mortality in the bivariate logistic regression analysis with *p*-value < 0.05 were included in the multivariate logistic regression analysis. The strength of association was determined using odds ratio and 95% confidence level. Statistical significance was stated at *P* value of < 0.05.

## Results

### Socio demographic data and risk factors of neonatal mortality

Out of 410 selected patient charts 391 neonatal charts were studied. The rest were discarded due to incompleteness. From all 275 (70.3%) were neonates less than 7 days and 209 (53.5%) were males. Regarding to the birth weight, 297 (76%) were between 2001 and 4000 kg. Most (76.5%) of the neonates were delivered between 37 and 42 weeks of gestation. Among all neonates, 241 (61.6%) were initiated breast milk within 1 hour of birth. From total 251 (64.9%) neonates were on exclusive breastfeeding. About three-fourths of the mothers 384 (72.6) had four or more ante natal care during their pregnancy. Majority of (96.5%) the mothers delivered in the health institution and 253 (64.7%) delivered normally through the vagina. Sepsis (23.8%), preterm (12.5%), pneumonia (10%) were the main recorded problems of the neonate during admission and from all 13.29% of admitted neonates were died (Table 1).

**Table 1:** Risk factors affecting neonatal mortality in Felege Hiwot referral Hospital, Bahir Dar, Amhara Regional State, North West Ethiopia 2016

Variables	Response	Frequency	Percent
Age	< 7 days	275	70.3
	8–28 days	116	29.7
Sex	Male	209	53.5
	female	182	46.5
Weight at birth	< 1500 kg	15	3.8
	1501–2000 kg	69	17.6
	2001–3999 kg	297	76.0
	> 4000 kg	10	2.6
Origin	Rural	151	38.6
	urban	240	61.4
Week of delivery	37–42 weeks	299	76.5
	< 37 weeks	63	16.1
	> 42 weeks	29	7.4
Breast feeding initiation time	Within 30 min	90	23.0
	With in 1 h	151	38.6
	After one hour	150	38.4
Exclusive breast feeding	Yes	254	64.9
	No	137	35.1
Immunization status	Yes	380	97.2
	No	11	2.8
Number of ANC	One	2	0.5
	Two	17	4.3
	Three	87	22.2
	Four	238	60.8
	Above four	46	11.8
Place of delivery	Health center	377	96.4
	home	14	4.6
Mode of delivery	Medical tool	118	30.3
	Operation	20	17.9
	Spontaneous	253	64.7
Congenital abnormality	Yes	3	0.8
	No	388	99.2
Chronic disease	Yes	15	3.8
	No	376	96.2
Problem after delivery	Pneumonia	39	10
	Sepsis	93	23.8
	CHF	9	3
	Jaundice	18	3
	Preterm/low birth wt	49	17
	Other (eg.DM, etc)	183	18
	CHD	9	2.3
	Jaundice	18	4.6
	Preterm/LBW	49	12.5
	Others	183	46.8
Chronic disease of mother	Yes	23	5.9
	No	368	94.1

### Factors associated with neonatal mortality

The associations of the independent and dependent variables were first tested by using bivariate analysis. Variables which were associated (*p <* 0.05) in the bivariate analysis were tested in the final multivariate analysis to see their significant association with neonatal mortality. The identified independent predictor of neonatal mortality were: early age of the newborn, gestational age < 37 weeks or preterm, late initiation of breastfeeding, non exclusive breastfeeding and inadequate ante natal visit.

Early age of the newborn (neonates in the first week) was significantly associated with neonatal mortality in the study area. Neonatal mortality was significantly higher at early age (first week) of the neonates than at later age [AOR = 0.39 (0.16–0.97)].

On the other hand gestational age at delivery was also significantly associated with the of neonatal mortality. The newborns who were preterm were two times higher to die than who were delivered term [AOR = 2.14 (1.0–4.52)].

Late initiation of breastfeeding was also associated with the occurrence of neonatal mortality. Mothers who initiate breastfeeding within 1 hour of birth of infant were almost three times higher to save their newborn than those who delayed breastfeeding initiation [AOR = 2.89 (0.99–8.38)].

Exclusive breastfeeding practice is known to save the life of a newborn. An infant who was not on exclusive breastfeeding was almost seven times higher to die than a neonate who was on exclusive breastfeeding [AOR = 6.77 (3.04–15.07)].

The number of ante natal vist was also associated with the prevalence of neonatal mortality. A neonate born from a mother with inadequate ante natal visit less than four times were almost five times higher to die than an infant born from a mother having adequate ante natal follow up [AOR = 5.02 (1.02–24.70)] (Table 2).

**Table 2:** Factors affecting neonatal mortality in Felege Hiwot referral Hospital, Bahir Dar, Amhara Regional State, North West Ethiopia 2016

Variables	Response	Number of neonate	Number of deaths	Prevalence % (95% CI)	Odds ratio (95% CI)	*P*-value
Age	Early	275	43	15.63(11.32–19.95)	0.39(0.16–0.97)	0.04
	Late	116	9	7.76(2.85–12.66)	1	
Week of delivery	37- 38 weeks	299	31	10.37(6.89–13.84)	1	–
	< 37 weeks	63	21	33.33(21.56–45.10)	2.14(1.0–4.52)	0.04
	> 42 weeks	29	0	–	–	–
Breast feeding initiation time	Early	90	5	5.56(0.78–10.33)	1	–
	Timely	151	14	9.27(4.62–13.93)	1.23(0.39–3.90)	0.73
	Lately	150	33	22.0(15.33–28.67)	2.89(0.99–8.38)	0.04
Exclusive breastfeeding	Yes	254	18	7.08(3.92–10.26)	6.77(3.04–15.07)	0.00
	No	137	34	24.82(17.53–32.09)	1	
Number of ANC visit	One	2	0	–	1	–
	Two	17	4	23.53(2.68–44.38)	6.48(0,89–46.69)	0.06
	Three	87	14	15.91(8.19–23.62)	2.35(0.45–12.26)	0.31
	Four	238	32	13.44(9.09–17.80)	5.02(1.02–24.70)	0.04
	More than four	46	2	4.35(−1.6–10.32)	–	

1 = references, *p* < .0.05 indicates significant variables

## Discussion

The prevalence of neonatal mortality in Felege Hiwot referral hospital was 13.29%. This finding was higher than the prevalence reported in north Gonder [9] and Jimma zone Ethiopia [5]. The variations may be due to methodological differences among studies and dissimilarity in socio cultural, health service utilization and economical variations among study participants of the study areas.

Risk factors like chronic disease of the mother, problems after delivery, weight of the newborn and type of aid during delivery were the risk factors considered by other researchers [4, 9, 10]. However, in the current study: age of neonate less than 7 days, week of delivery, initiation of breast feeding, non exclusive breastfeeding and inadequate number of ANC visit were significantly associated with neonatal mortality.

Early age (< 7 days) of the newborn and being preterm were significantly associated with neonatal mortality. The occurrence of neonatal death was higher in preterm’s than those born to term. Which consistent with studies [8, 10–16]. This is due to the fact that being preterm exposes the new born for different conditions. Since they have many physiologic challenges to adapt extra uterine life. Due to this they are exposed to different fatal conditions like: hypothermia, respiratory center depression, different cardiovascular and hematological conditions like anemia, hyperbilirubinemia, immature immune defences which exposes them to infections, nutritional and gastrointestinal problems like poor feeding and entrocollitis, metabolic problems like hypoglycemia, fluid and electrolyte imbalance, low glomerular filtration rate and inability to handle water and solute loss are the major problems associated with preterm that increases the incidence of neonatal mortality.

Late initiation of breast feeding and non exclusive breastfeeding were the determinant factor for neonatal mortality. Neonatal mortality was higher in neonates who started breastfeeding after one hour and in those who were not on exclusive breastfeeding. Which is in line with studies done [5, 9]. This due to breast milk is the ideal nutrient for the newborn, easily digestible absorbable and metabolized, promote bonding, improved behavioural and neurodevelopment, protects against various infectious diseases and promotes long term health which ultimately decreases neonatal mortality if it is practiced optimally.

Neonatal mortality was significantly associated with inadequate number of ante natal care visits. The danger of neonatal mortality was significantly reduced in those mothers who performed ANC visit four times and above than those who had less than four ANC visits. This is similar with the previous studies [9, 17, 18]. This may be due to proper ante natal vist increases early detection and management of the problems related with the pregnancy which ultimately improves the neonatal outcome.

Among the common diseases identified as a causes of neonatal mortality: pneumonia (5.13%), sepsis (9.68), congestive heart failure (33.33), jaundice (16.67), premature delivery (34.69) and other unidentified causes (9.84%). Which is consistent with studies [9, 11, 14–16] where the most common conditions for neonatal mortalities were preterm, asphyxia, neonatal infections; diarrhoea, sepsis, pneumonia, tetanus, and congenital malformations.

## Conclusions

This study stated that the level of neonatal mortality is high in the study area. The great majority of neonatal deaths occurred in the first week of life, in neonates born preterm, not started breastfeeding on time, not on exclusive breastfeeding and in those mothers did not have adequate ante natal care visit. Recommendations to decrease neonatal mortality were: ensuring ante natal care during pregnancy, proper delivery care, and immediate postnatal care, increasing ante natal visits, delivering in health facility, providing comprehensive neonatal care, prevention and interventions of neonatal infection were recommended to reduce neonatal mortality.

### Limitations

The study was identified based on the documented data and could not display all factors that are not documented in the patient’s files, representativeness, completeness and quality of the recorded information.

## Abbreviations

ANC: Anti natal care
DHS: Demographic and health survey
FHRH: Felege Hiwot Referral Hospital
ICU: Intensive care unit
MDG: Millennium development goal
NMR: Neonatal mortality rate
PROM: Premature rupture of membrane
SPSS: Statistical package soft ware
WHO: World Health Organization
